# Poly[[(μ_3_-2,4,6-tri-4-pyridyl-1,3,5-triazine)copper(I)] nitrate monohydrate]

**DOI:** 10.1107/S1600536811011445

**Published:** 2011-03-31

**Authors:** Miao Feng, Hui-Juan Tian, Huai-Feng Mi, Tong-Liang Hu

**Affiliations:** a Biochemical Section of Key Laboratory of Functional Polymer Materials, Ministry of Education of China, Chemical School of Nankai University, 300071 Tianjin, People’s Republic of China; b Department of Chemistry, Nankai University, Tianjin 300071, People’s Republic of China

## Abstract

In the title compound, {[Cu(C_18_H_12_N_6_)]NO_3_·H_2_O}_*n*_, the Cu^I^ ion is coordinated by three N atoms [Cu—N 1.962 (3)–2.019 (3) Å] from three 2,4,6-tri-4-pyridyl-1,3,5-triazine (*L*) ligands. Each *L* ligand bridges three Cu^I^ atoms, generating a positively charged three-dimensional polymeric network with voids propagated along the *b* axis. These voids are filled with NO_3_^−^ anions with a shortest Cu⋯O distance of 2.645 (3) Å and water mol­ecules, which are linked into negatively charged helical chains *via* inter­molecular O—H⋯O hydrogen bonds.

## Related literature

For metal complexes with 2,4,6-tri­(4-pyrid­yl)-1,3,5-triazine ligands, see: Abrahams *et al.* (1999 ▶); Dybtsev *et al.* (2004 ▶); Barrios *et al.* (2007 ▶).
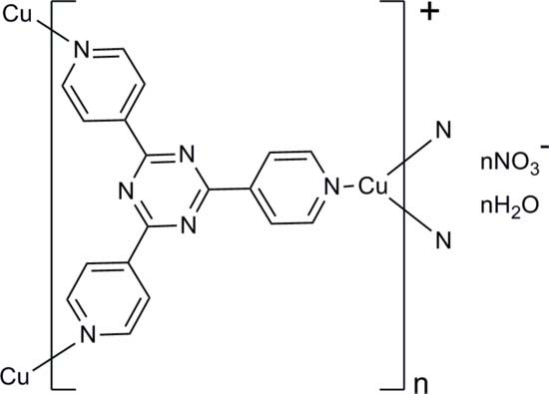


## Experimental

### 

#### Crystal data

[Cu(C_18_H_12_N_6_)]NO_3_·H_2_O
*M* *_r_* = 455.90
Monoclinic, 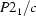

*a* = 9.917 (2) Å
*b* = 8.7409 (17) Å
*c* = 22.499 (6) Å
β = 107.43 (3)°
*V* = 1860.7 (7) Å^3^
*Z* = 4
Mo *K*α radiation
μ = 1.22 mm^−1^
*T* = 293 K
0.10 × 0.10 × 0.10 mm

#### Data collection

Rigaku SCX-mini diffractometer
Absorption correction: multi-scan (*ABSCOR*; Higashi, 1995 ▶) *T*_min_ = 0.736, *T*_max_ = 1.000
18394 measured reflections
4262 independent reflections
2717 reflections with *I* > 2σ(*I*)
*R*_int_ = 0.097

#### Refinement

*R*[*F* ^2^ > 2σ(*F* ^2^)] = 0.070
*wR*(*F* ^2^) = 0.125
*S* = 1.09
4262 reflections
271 parameters
H-atom parameters constrained
Δρ_max_ = 0.36 e Å^−3^
Δρ_min_ = −0.35 e Å^−3^

### 

Data collection: *PROCESS-AUTO* (Rigaku, 1998 ▶); cell refinement: *PROCESS-AUTO*; data reduction: *CrystalStructure* (Rigaku/MSC, 2002 ▶); program(s) used to solve structure: *SHELXS97* (Sheldrick, 2008 ▶); program(s) used to refine structure: *SHELXL97* (Sheldrick, 2008 ▶); molecular graphics: *SHELXTL* (Sheldrick, 2008 ▶); software used to prepare material for publication: *publCIF* (Westrip, 2010 ▶).

## Supplementary Material

Crystal structure: contains datablocks I, global. DOI: 10.1107/S1600536811011445/cv5064sup1.cif

Structure factors: contains datablocks I. DOI: 10.1107/S1600536811011445/cv5064Isup2.hkl

Additional supplementary materials:  crystallographic information; 3D view; checkCIF report

## Figures and Tables

**Table 1:** Hydrogen-bond geometry (Å, °)

*D*—H⋯*A*	*D*—H	H⋯*A*	*D*⋯*A*	*D*—H⋯*A*
O4*W*—H4*WA*⋯O2	0.91	2.23	3.057 (7)	151
O4*W*—H4*WB*⋯O2^i^	0.92	2.23	3.082 (7)	155

Symmetry code: (i) 
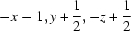
.

**Fig. 1.**
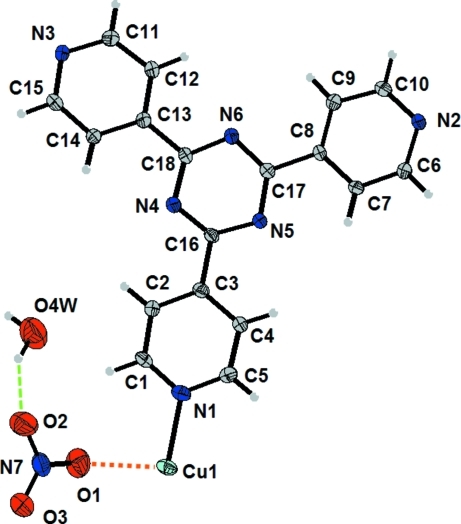
A content of the asymmetric unit of 1 showing the atomic numbering and displacement ellipsoids drawn at the 30% probability level. The O—H···O hydrogen bond is shown by the dashed green line, and the shortest Cu···O distance is shown by the dotted red line.

## supplementary crystallographic information

### Comment

As an interesting polydentate nitrogen donor ligand, 2,4,6-tris(4-pyridyl)-1,3,5-triazine(*L*) has attracted increasing attention in the synthesis of novel transition metal complexes with novel topology and properties (Abrahams *et al.* 1999; Dybtsev *et al.* 2004; Barrios *et al.* 2007). Our interest in 2,4,6-tris(4-pyridyl)-1,3,5-triazine transition metal complexes prompts us to report here the crystal structure of the title compound (1).

In 1 (Fig. 1), each Cu^I^ ion is coordinated by three N atoms [Cu—N 1.962 (3)–2.019 (3) Å] from three ligands *L*, and each ligand *L* bridge three Cu^I^ centers generating positively charged three-dimensional polymeric network with the voids propagated along axis *b*. These voids are filled with NO_3_^-^ anions with the shortest Cu···O distance of 2.645 (3) Å and crystalline water molecules, which are linked into negatively charged helical chains *via* intermolecular O—H···O hydrogen bonds.

### Experimental

In a typical synthesis, a mixture of Cu(NO_3_)_2_.6H_2_O (1 mmol), 2,4,6-tris(4-pyridyl)-1,3,5-triazine (1 mmol) and methanol (10 ml), was added to a 20 ml Teflon-lined reactor under autogenous pressure at 140 °C for 3 days.

### Refinement

C-bound H atoms were positioned geometrically (C—H = 0.93 Å) and allowed to ride on their parent atoms, with *U*_iso_(H) = 1.2*U*_eq_(C). The H atoms of the water molecules were located on a difference map, and refined as riding in their as-found relative positions with *U*_iso_(H) = 1.2*U*_eq_(O).

### Crystal data

**Table:**

[Cu(C_18_H_12_N_6_)]NO_3_·H_2_O	*F*(000) = 928
*M**_r_* = 455.90	*D*_x_ = 1.627 Mg m^−^^3^
Monoclinic, *P*2_1_/*c*	Mo *K*α radiation, λ = 0.71073 Å
Hall symbol: -P 2ybc	Cell parameters from 14882 reflections
*a* = 9.917 (2) Å	θ = 3.0–27.7°
*b* = 8.7409 (17) Å	µ = 1.22 mm^−^^1^
*c* = 22.499 (6) Å	*T* = 293 K
β = 107.43 (3)°	Block, red
*V* = 1860.7 (7) Å^3^	0.10 × 0.10 × 0.10 mm
*Z* = 4	

### Data collection

**Table:**

Rigaku SCX-mini diffractometer	4262 independent reflections
Radiation source: fine-focus sealed tube	2717 reflections with *I* > 2σ(*I*)
graphite	*R*_int_ = 0.097
ω scans	θ_max_ = 27.5°, θ_min_ = 3.1°
Absorption correction: multi-scan (*ABSCOR*; Higashi, 1995)	*h* = −12→12
*T*_min_ = 0.736, *T*_max_ = 1.000	*k* = −11→11
18394 measured reflections	*l* = −28→29

### Refinement

**Table:**

Refinement on *F*^2^	Primary atom site location: structure-invariant direct methods
Least-squares matrix: full	Secondary atom site location: difference Fourier map
*R*[*F*^2^ > 2σ(*F*^2^)] = 0.070	Hydrogen site location: inferred from neighbouring sites
*wR*(*F*^2^) = 0.125	H-atom parameters constrained
*S* = 1.09	*w* = 1/[σ^2^(*F*_o_^2^) + (0.0335*P*)^2^ + 2.1056*P*] where *P* = (*F*_o_^2^ + 2*F*_c_^2^)/3
4262 reflections	(Δ/σ)_max_ < 0.001
271 parameters	Δρ_max_ = 0.36 e Å^−^^3^
0 restraints	Δρ_min_ = −0.35 e Å^−^^3^

### Special details

**Table:**

Geometry. All e.s.d.'s (except the e.s.d. in the dihedral angle between two l.s. planes) are estimated using the full covariance matrix. The cell e.s.d.'s are taken into account individually in the estimation of e.s.d.'s in distances, angles and torsion angles; correlations between e.s.d.'s in cell parameters are only used when they are defined by crystal symmetry. An approximate (isotropic) treatment of cell e.s.d.'s is used for estimating e.s.d.'s involving l.s. planes.
Refinement. Refinement of *F*^2^ against ALL reflections. The weighted *R*-factor *wR* and goodness of fit *S* are based on *F*^2^, conventional *R*-factors *R* are based on *F*, with *F* set to zero for negative *F*^2^. The threshold expression of *F*^2^ > σ(*F*^2^) is used only for calculating *R*-factors(gt) *etc*. and is not relevant to the choice of reflections for refinement. *R*-factors based on *F*^2^ are statistically about twice as large as those based on *F*, and *R*- factors based on ALL data will be even larger.

### Fractional atomic coordinates and isotropic or equivalent isotropic displacement parameters (Å^2^)

**Table:**

	*x*	*y*	*z*	*U*_iso_*/*U*_eq_	
C1	−0.1691 (5)	0.5154 (5)	0.27731 (19)	0.0411 (12)	
H1	−0.2584	0.5537	0.2569	0.049*	
C2	−0.1040 (4)	0.5629 (5)	0.33689 (19)	0.0380 (11)	
H2	−0.1487	0.6318	0.3563	0.046*	
C3	0.0293 (4)	0.5073 (5)	0.36812 (18)	0.0296 (9)	
C4	0.0904 (4)	0.4047 (5)	0.33735 (18)	0.0344 (10)	
H4	0.1795	0.3642	0.3568	0.041*	
C5	0.0177 (4)	0.3635 (5)	0.27772 (19)	0.0383 (11)	
H5	0.0600	0.2945	0.2573	0.046*	
C6	0.5691 (4)	0.2273 (5)	0.55716 (19)	0.0379 (11)	
H6	0.5981	0.1474	0.5366	0.046*	
C7	0.4531 (4)	0.3104 (5)	0.52532 (18)	0.0321 (10)	
H7	0.4053	0.2872	0.4840	0.039*	
C8	0.4077 (4)	0.4293 (5)	0.55511 (18)	0.0276 (9)	
C9	0.4827 (4)	0.4592 (5)	0.61641 (19)	0.0398 (11)	
H9	0.4557	0.5381	0.6382	0.048*	
C10	0.5983 (4)	0.3696 (5)	0.64455 (19)	0.0407 (11)	
H10	0.6482	0.3904	0.6858	0.049*	
C11	0.0496 (4)	0.9682 (5)	0.6342 (2)	0.0388 (11)	
H11	0.0994	1.0111	0.6722	0.047*	
C12	0.1191 (4)	0.8664 (5)	0.60714 (18)	0.0354 (10)	
H12	0.2124	0.8393	0.6271	0.042*	
C13	0.0474 (4)	0.8050 (5)	0.54967 (17)	0.0270 (9)	
C14	−0.0906 (4)	0.8507 (5)	0.52211 (18)	0.0310 (10)	
H14	−0.1408	0.8143	0.4829	0.037*	
C15	−0.1526 (4)	0.9498 (5)	0.55297 (19)	0.0360 (10)	
H15	−0.2462	0.9775	0.5342	0.043*	
C16	0.1021 (4)	0.5523 (5)	0.43321 (17)	0.0281 (9)	
C17	0.2806 (4)	0.5165 (5)	0.52141 (18)	0.0276 (9)	
C18	0.1122 (4)	0.6898 (5)	0.51893 (18)	0.0278 (9)	
Cu1	−0.19856 (5)	0.38069 (7)	0.15691 (2)	0.03757 (18)	
N1	−0.1108 (4)	0.4169 (4)	0.24703 (15)	0.0364 (9)	
N2	0.6432 (3)	0.2554 (4)	0.61653 (15)	0.0321 (8)	
N3	−0.0852 (3)	1.0091 (4)	0.60904 (15)	0.0329 (8)	
N4	0.0422 (3)	0.6577 (4)	0.45964 (15)	0.0313 (8)	
N5	0.2221 (3)	0.4792 (4)	0.46175 (14)	0.0290 (8)	
N6	0.2306 (3)	0.6215 (4)	0.55228 (14)	0.0308 (8)	
N7	−0.4795 (5)	0.6084 (6)	0.1312 (2)	0.0619 (12)	
O1	−0.3565 (4)	0.6301 (5)	0.13251 (19)	0.0806 (12)	
O2	−0.5122 (4)	0.6217 (6)	0.18018 (19)	0.1031 (17)	
O3	−0.5691 (4)	0.5721 (6)	0.0832 (2)	0.0948 (15)	
O4W	−0.3407 (5)	0.8630 (5)	0.2697 (2)	0.1142 (17)	
H4WA	−0.4160	0.8171	0.2417	0.142*	
H4WB	−0.3653	0.9580	0.2804	0.142*	

### Atomic displacement parameters (Å^2^)

**Table:**

	*U*^11^	*U*^22^	*U*^33^	*U*^12^	*U*^13^	*U*^23^
C1	0.033 (2)	0.058 (3)	0.026 (2)	0.007 (2)	−0.002 (2)	0.001 (2)
C2	0.031 (2)	0.047 (3)	0.034 (2)	0.010 (2)	0.006 (2)	−0.007 (2)
C3	0.029 (2)	0.033 (2)	0.024 (2)	−0.0004 (19)	0.0058 (18)	0.0032 (18)
C4	0.030 (2)	0.041 (3)	0.029 (2)	0.008 (2)	0.0042 (19)	−0.002 (2)
C5	0.043 (3)	0.041 (3)	0.031 (2)	0.005 (2)	0.011 (2)	−0.005 (2)
C6	0.036 (2)	0.040 (3)	0.033 (2)	0.014 (2)	0.005 (2)	−0.006 (2)
C7	0.033 (2)	0.035 (3)	0.023 (2)	0.0064 (19)	0.0021 (19)	−0.0029 (18)
C8	0.022 (2)	0.031 (2)	0.027 (2)	0.0049 (17)	0.0033 (17)	0.0016 (18)
C9	0.040 (3)	0.041 (3)	0.034 (2)	0.015 (2)	0.005 (2)	−0.007 (2)
C10	0.040 (3)	0.047 (3)	0.028 (2)	0.011 (2)	−0.001 (2)	−0.005 (2)
C11	0.035 (2)	0.045 (3)	0.034 (2)	0.002 (2)	0.006 (2)	−0.010 (2)
C12	0.026 (2)	0.042 (3)	0.035 (2)	0.006 (2)	0.0054 (19)	−0.002 (2)
C13	0.026 (2)	0.027 (2)	0.028 (2)	0.0003 (17)	0.0086 (18)	0.0022 (18)
C14	0.029 (2)	0.032 (3)	0.029 (2)	0.0042 (19)	0.0052 (18)	−0.0044 (19)
C15	0.029 (2)	0.043 (3)	0.032 (2)	0.003 (2)	0.001 (2)	−0.001 (2)
C16	0.027 (2)	0.029 (2)	0.026 (2)	0.0028 (18)	0.0038 (18)	0.0024 (18)
C17	0.026 (2)	0.028 (2)	0.027 (2)	−0.0003 (18)	0.0060 (18)	0.0015 (18)
C18	0.024 (2)	0.028 (2)	0.029 (2)	0.0002 (17)	0.0048 (18)	0.0023 (18)
Cu1	0.0337 (3)	0.0470 (4)	0.0281 (3)	−0.0141 (3)	0.0034 (2)	−0.0025 (3)
N1	0.037 (2)	0.044 (2)	0.0250 (18)	−0.0085 (17)	0.0054 (16)	0.0013 (16)
N2	0.0297 (19)	0.035 (2)	0.0277 (18)	0.0108 (16)	0.0024 (16)	0.0016 (16)
N3	0.0270 (19)	0.037 (2)	0.034 (2)	0.0079 (16)	0.0077 (16)	−0.0036 (17)
N4	0.0312 (19)	0.032 (2)	0.0281 (18)	0.0071 (15)	0.0048 (16)	0.0006 (15)
N5	0.0273 (18)	0.032 (2)	0.0249 (18)	0.0054 (15)	0.0038 (15)	−0.0011 (15)
N6	0.0275 (17)	0.032 (2)	0.0303 (18)	0.0049 (17)	0.0052 (15)	−0.0014 (17)
N7	0.044 (3)	0.080 (4)	0.058 (3)	0.017 (3)	0.010 (2)	0.012 (3)
O1	0.054 (2)	0.097 (3)	0.097 (3)	−0.008 (2)	0.032 (2)	0.008 (3)
O2	0.064 (3)	0.190 (5)	0.060 (3)	−0.006 (3)	0.025 (2)	0.000 (3)
O3	0.068 (3)	0.131 (4)	0.071 (3)	0.022 (3)	−0.002 (2)	−0.021 (3)
O4W	0.088 (3)	0.100 (4)	0.130 (4)	0.007 (3)	−0.005 (3)	−0.021 (3)

### Geometric parameters (Å, °)

**Table:**

C1—N1	1.332 (5)	C12—C13	1.384 (5)
C1—C2	1.367 (5)	C12—H12	0.9300
C1—H1	0.9300	C13—C14	1.381 (5)
C2—C3	1.386 (5)	C13—C18	1.473 (5)
C2—H2	0.9300	C14—C15	1.366 (5)
C3—C4	1.380 (5)	C14—H14	0.9300
C3—C16	1.479 (5)	C15—N3	1.342 (5)
C4—C5	1.367 (5)	C15—H15	0.9300
C4—H4	0.9300	C16—N4	1.329 (5)
C5—N1	1.338 (5)	C16—N5	1.334 (5)
C5—H5	0.9300	C17—N6	1.332 (5)
C6—N2	1.340 (5)	C17—N5	1.334 (5)
C6—C7	1.368 (5)	C18—N6	1.331 (5)
C6—H6	0.9300	C18—N4	1.336 (5)
C7—C8	1.383 (5)	Cu1—N2^i^	1.962 (3)
C7—H7	0.9300	Cu1—N1	1.978 (3)
C8—C9	1.382 (5)	Cu1—N3^ii^	2.019 (3)
C8—C17	1.474 (5)	N2—Cu1^iii^	1.962 (3)
C9—C10	1.376 (5)	N3—Cu1^iv^	2.019 (3)
C9—H9	0.9300	N7—O3	1.218 (5)
C10—N2	1.326 (5)	N7—O1	1.226 (5)
C10—H10	0.9300	N7—O2	1.243 (5)
C11—N3	1.335 (5)	O4W—H4WA	0.9125
C11—C12	1.374 (6)	O4W—H4WB	0.9175
C11—H11	0.9300		
			
N1—C1—C2	123.3 (4)	C14—C13—C18	120.0 (3)
N1—C1—H1	118.4	C12—C13—C18	122.0 (3)
C2—C1—H1	118.4	C15—C14—C13	119.4 (4)
C1—C2—C3	119.3 (4)	C15—C14—H14	120.3
C1—C2—H2	120.4	C13—C14—H14	120.3
C3—C2—H2	120.4	N3—C15—C14	123.3 (4)
C4—C3—C2	118.0 (4)	N3—C15—H15	118.3
C4—C3—C16	120.8 (3)	C14—C15—H15	118.3
C2—C3—C16	121.2 (4)	N4—C16—N5	124.8 (3)
C5—C4—C3	118.8 (4)	N4—C16—C3	118.4 (3)
C5—C4—H4	120.6	N5—C16—C3	116.7 (4)
C3—C4—H4	120.6	N6—C17—N5	125.1 (3)
N1—C5—C4	123.7 (4)	N6—C17—C8	118.8 (3)
N1—C5—H5	118.1	N5—C17—C8	116.0 (4)
C4—C5—H5	118.1	N6—C18—N4	125.1 (4)
N2—C6—C7	123.3 (4)	N6—C18—C13	118.5 (3)
N2—C6—H6	118.4	N4—C18—C13	116.3 (3)
C7—C6—H6	118.4	N2^i^—Cu1—N1	128.12 (14)
C6—C7—C8	119.4 (4)	N2^i^—Cu1—N3^ii^	122.55 (14)
C6—C7—H7	120.3	N1—Cu1—N3^ii^	109.03 (14)
C8—C7—H7	120.3	C1—N1—C5	116.9 (3)
C9—C8—C7	118.0 (4)	C1—N1—Cu1	120.2 (3)
C9—C8—C17	122.5 (4)	C5—N1—Cu1	122.1 (3)
C7—C8—C17	119.5 (3)	C10—N2—C6	116.7 (3)
C10—C9—C8	118.5 (4)	C10—N2—Cu1^iii^	124.9 (3)
C10—C9—H9	120.8	C6—N2—Cu1^iii^	118.3 (3)
C8—C9—H9	120.8	C11—N3—C15	116.6 (4)
N2—C10—C9	124.2 (4)	C11—N3—Cu1^iv^	123.3 (3)
N2—C10—H10	117.9	C15—N3—Cu1^iv^	119.1 (3)
C9—C10—H10	117.9	C16—N4—C18	115.1 (3)
N3—C11—C12	123.9 (4)	C17—N5—C16	115.1 (3)
N3—C11—H11	118.1	C18—N6—C17	114.7 (3)
C12—C11—H11	118.1	O3—N7—O1	121.1 (5)
C11—C12—C13	118.6 (4)	O3—N7—O2	119.7 (5)
C11—C12—H12	120.7	O1—N7—O2	119.1 (5)
C13—C12—H12	120.7	H4WA—O4W—H4WB	110.6
C14—C13—C12	118.0 (4)		
			
N1—C1—C2—C3	0.1 (7)	C12—C13—C18—N4	−169.4 (4)
C1—C2—C3—C4	−0.4 (6)	C2—C1—N1—C5	0.2 (7)
C1—C2—C3—C16	−178.4 (4)	C2—C1—N1—Cu1	−170.1 (3)
C2—C3—C4—C5	0.4 (6)	C4—C5—N1—C1	−0.1 (6)
C16—C3—C4—C5	178.4 (4)	C4—C5—N1—Cu1	169.9 (3)
C3—C4—C5—N1	−0.2 (7)	N2^i^—Cu1—N1—C1	−82.3 (4)
N2—C6—C7—C8	−0.4 (7)	N3^ii^—Cu1—N1—C1	103.9 (3)
C6—C7—C8—C9	0.2 (6)	N2^i^—Cu1—N1—C5	107.9 (3)
C6—C7—C8—C17	−178.5 (4)	N3^ii^—Cu1—N1—C5	−65.8 (4)
C7—C8—C9—C10	0.0 (6)	C9—C10—N2—C6	−0.3 (7)
C17—C8—C9—C10	178.6 (4)	C9—C10—N2—Cu1^iii^	−176.6 (4)
C8—C9—C10—N2	0.1 (7)	C7—C6—N2—C10	0.5 (7)
N3—C11—C12—C13	1.8 (7)	C7—C6—N2—Cu1^iii^	177.0 (3)
C11—C12—C13—C14	0.6 (6)	C12—C11—N3—C15	−2.5 (7)
C11—C12—C13—C18	−177.1 (4)	C12—C11—N3—Cu1^iv^	165.7 (3)
C12—C13—C14—C15	−2.2 (6)	C14—C15—N3—C11	0.8 (6)
C18—C13—C14—C15	175.6 (4)	C14—C15—N3—Cu1^iv^	−167.9 (3)
C13—C14—C15—N3	1.5 (7)	N5—C16—N4—C18	0.7 (6)
C4—C3—C16—N4	176.4 (4)	C3—C16—N4—C18	178.4 (3)
C2—C3—C16—N4	−5.6 (6)	N6—C18—N4—C16	−2.4 (6)
C4—C3—C16—N5	−5.7 (6)	C13—C18—N4—C16	−179.3 (3)
C2—C3—C16—N5	172.2 (4)	N6—C17—N5—C16	−2.1 (6)
C9—C8—C17—N6	−4.5 (6)	C8—C17—N5—C16	175.8 (3)
C7—C8—C17—N6	174.0 (4)	N4—C16—N5—C17	1.3 (6)
C9—C8—C17—N5	177.5 (4)	C3—C16—N5—C17	−176.4 (3)
C7—C8—C17—N5	−4.0 (6)	N4—C18—N6—C17	1.7 (6)
C14—C13—C18—N6	−164.3 (4)	C13—C18—N6—C17	178.6 (3)
C12—C13—C18—N6	13.4 (6)	N5—C17—N6—C18	0.7 (6)
C14—C13—C18—N4	12.9 (5)	C8—C17—N6—C18	−177.1 (4)

Symmetry codes: (i) *x*−1, −*y*+1/2, *z*−1/2; (ii) *x*, −*y*+3/2, *z*−1/2; (iii) *x*+1, −*y*+1/2, *z*+1/2; (iv) *x*, −*y*+3/2, *z*+1/2.

### Hydrogen-bond geometry (Å, °)

**Table:**

*D*—H···*A*	*D*—H	H···*A*	*D*···*A*	*D*—H···*A*
O4W—H4WA···O2	0.91	2.23	3.057 (7)	151
O4W—H4WB···O2^v^	0.92	2.23	3.082 (7)	155

Symmetry codes:  (v) −*x*−1, *y*+1/2, −*z*+1/2.
